# Mapping T Cell Responses to Native and Neo-Islet Antigen Epitopes in at Risk and Type 1 Diabetes Subjects

**DOI:** 10.3389/fimmu.2021.675746

**Published:** 2021-06-25

**Authors:** Sefina Arif, Irma Pujol-Autonell, Yogesh Kamra, Evangelia Williams, Norkhairin Yusuf, Clara Domingo-Vila, Yasaman Shahrabi, Emily Pollock, Leena Khatri, Mark Peakman, Timothy Tree, Anna Lorenc

**Affiliations:** Department of Immunobiology, King’s College London, Guy’s Hospital, London, United Kingdom

**Keywords:** neoepitopes, T cells, cytokines, T cell receptor, genes, type 1 diabetes, autoantibody, transcriptome

## Abstract

**Aims:**

Recent studies highlight the potentially important role of neoepitopes in breaking immune tolerance in type 1 diabetes. T cell reactivity to these neoepitopes has been reported, but how this response compares quantitatively and phenotypically with previous reports on native epitopes is not known. Thus, an understanding of the relationship between native and neoepitopes and their role as tolerance breakers or disease drivers in type 1 diabetes is required. We set out to compare T cell reactivity and phenotype against a panel of neo- and native islet autoantigenic epitopes to examine how this relates to stages of type 1 diabetes development.

**Methods:**

Fifty-four subjects comprising patients with T1D, and autoantibody-positive unaffected family members were tested against a panel of neo- and native epitopes by ELISPOT (IFN-*γ*, IL-10, and IL-17). A further subset of two patients was analyzed by Single Cell Immune Profiling (RNAseq and TCR *α*/*β*) after stimulation with pools of native and neoepitope peptides.

**Results:**

T cell responses to native and neoepitopes were present in patients with type 1 diabetes and at-risk subjects, and overall, there were no significant differences in the frequency, magnitude, or phenotype between the two sets of peptide stimuli. Single cell RNAseq on responder T cells revealed a similar profile in T1D patients stimulated with either neo- or native epitopes. A pro-inflammatory gene expression profile (TNF-α, IFN-*γ*) was dominant in both native and neoepitope stimulated T cells. TCRs with identical clonotypes were found in T cell responding to both native and neoepitopes.

**Conclusion/Interpretation:**

These data suggest that in peripheral blood, T cell responses to both native and neoepitopes are similar in terms of frequency and phenotype in patients with type 1 diabetes and high-risk unaffected family members. Furthermore, using a combination of transcriptomic and clonotypic analyses, albeit using a limited panel of peptides, we show that neoepitopes are comparable to native epitopes currently in use for immune-monitoring studies.

## Introduction

Type 1 diabetes arises as a result of immune-mediated loss of *β* cell mass and function. A lack of self-tolerance against islet autoantigens facilitates this destruction; one of the hallmarks of type 1 diabetes pathogenesis is the autoantigen-specific clonal expansion of T cells (1). Upon stimulation with peptides of islet autoantigens, these T cells respond and secrete cytokines such as interferon-*γ*, IL-10, and IL-17 which can be measured in subjects with type 1 diabetes and used to illustrate the heterogeneity of the disease (2–4) Furthermore, T cell responses elicited against peptides of proinsulin distinguish age-related heterogeneity in type 1 diabetes pathogenesis (4). Previous studies have suggested that T cell epitopes such as proinsulin peptide C13–32 elicit pro-inflammatory responses in approximately 70% of patients (4), and responses to this epitope can also be detected in autoantibody-positive unaffected family members (3).

Recent studies have highlighted the role of the *β* cell in orchestrating its own sustained attrition and demise by enabling a repertoire of non-germline encoded “neoepitopes” previously not encountered by the immune system (5).

Neoepitopes in T1D can be generated through several mechanisms including post-translational modification of peptides, for example *via* enzymatic deamidation or citrullination which can augment binding to HLA molecules and boost T cell responses (6); alternative RNA-splicing results in proteins being encoded from an alternative open reading frame (7) and peptide fusion of islet-derived peptides (8). Peptide fusion is a post-translational modification in which peptide fragments of proteins such as proinsulin are modified through fusion with peptides from other secretory granule proteins resulting in novel peptidic species termed hybrid insulin peptides (HIPs).

The fact that neoepitope specific T cells have been isolated from the islets of subjects with type 1 diabetes after death (8, 9) has provided a strong implication that these antigenic drivers and T cells directed against them could be relevant to disease pathology.

Similarly, T cell reactivity to neoepitopes such as modified glucose-regulated protein 78 (GRP78), HIPS and post-translationally modified GAD peptides has been demonstrated in the peripheral blood of subjects with type 1 diabetes and is observed in a greater frequency in these individuals compared to healthy donors (10–12).

In some cases, reactivity to neoepitopes has been shown to be more enhanced than that directed to the native epitopes (10, 12); for example, CD4 T cells directed against citrullinated GRP78 elicited T cell responses at a greater frequency in patients compared to the native epitope. Thus, growing evidence suggests that neoepitopes are relevant in type 1 diabetes pathology, but whether these are germane to the pathological response, are early drivers of disease or arise secondarily, and whether they serve as biomarkers remain issues to be addressed.

It is not clear how T cell responses to native and neoepitopes interlink and indeed which comes first; hence, an understanding of the relationship between native and neoepitopes and their role as tolerance breakers or disease drivers in type 1 diabetes is required. In order to gain this knowledge, both sets of epitopes need to be tested in parallel on the same patient cohorts.

In this study, we set out to evaluate T cell response to neoepitopes and established native epitopes in patients with type 1 diabetes and high-risk autoantibody-positive non-diabetic subjects to determine whether an epitope hierarchy exists. We then proceed to analyze T cells responding to native and neoepitopes at the single cell level and compare TCR usage to help address the phenotypic, transcriptomic, and clonal nature of the responses.

## Methods

### Subjects

Blood was obtained from 54 subjects, 41 of whom had type 1 diabetes, and 13 subjects comprised of autoantibody-positive unaffected family members (UFMs); both groups consisted of children (≤16 years of age) and adults (≥16 years of age). In the type 1 diabetes group there were 14 participants in the group comprising of children (disease duration <1.5 months; median age 11 years; nine males) and 27 in the adult group (disease duration <8 months; median age 24 years; 14 males). In the UFM, there were five children (median age 11; 5 males) and eight adults (median age 37; six males). Forty-six subjects were recruited through the INNODIA natural history study (13). HLA and autoantibody data on all the subjects are shown in **Table 1**.

**Table 1:** HLA and autoantibody status of subjects with type 1 diabetes and unaffected family members (UFM).

Subject	HLA-DR/DQ				AUTOANTIBODIES			
	DR4+	DQ8+	DR3+	DQ2+	IAA	GADA	IA2A	ZnT8A
								
ND01	N	N	Y	Y	NEG	POS	NEG	POS
ND02	Y	Y	Y	Y	POS	NEG	NEG	NEG
ND03	Y	Y	N	N	N/A	N/A	N/A	N/A
ND04	N	N	Y	Y	N/A	N/A	N/A	N/A
ND05	N	N	Y	Y	POS	POS	POS	POS
ND06	N	N	Y	Y	POS	POS	POS	POS
ND07	N	N	Y	Y	POS	POS	POS	NEG
ND08	N	N	N	N	POS	POS	POS	POS
ND09	N	N	N	N	POS	NEG	NEG	NEG
ND10	N	N	N	N	NEG	NEG	NEG	NEG
ND11	N	N	Y	Y	POS	POS	POS	POS
ND12	N	N	N	N	POS	NEG	POS	POS
ND13	N	N	N	N	POS	POS	POS	NEG
ND14	N	N	N	N	POS	NEG	NEG	POS
ND15	N	N	Y	Y	NEG	POS	POS	POS
ND16	N	N	N	N	POS	POS	POS	POS
ND17	Y	N	N	N	NEG	POS	POS	POS
ND18	Y	Y	N	N	POS	POS	POS	POS
ND19	Y	Y	N	N	POS	NEG	POS	POS
ND20	Y	Y	Y	Y	POS	POS	POS	POS
ND21	Y	Y	Y	Y	POS	POS	POS	POS
ND22	Y	Y	Y	Y	NEG	POS	NEG	POS
ND23	Y	Y	N	N	POS	POS	NEG	POS
ND24	Y	Y	Y	Y	NEG	NEG	NEG	NEG
ND25	Y	Y	Y	Y	NEG	POS	NEG	NEG
ND26	Y	Y	Y	Y	POS	NEG	NEG	NEG
ND27	Y	Y	Y	Y	POS	POS	NEG	NEG
ND28	Y	N	N	N	POS	POS	NEG	POS
ND29	Y	Y	Y	Y	NEG	POS	NEG	POS
ND30	Y	N	N	N	POS	NEG	NEG	NEG
ND31	Y	Y	N	N	POS	NEG	POS	NEG
ND32	Y	Y	N	N	POS	POS	POS	NEG
ND33	Y	Y	Y	Y	POS	POS	POS	POS
ND34	Y	Y	Y	Y	NEG	POS	POS	POS
ND35	Y	Y	Y	Y	POS	POS	POS	POS
ND36	N	N	Y	Y	NEG	POS	POS	NEG
ND37	Y	Y	N	N	NEG	POS	NEG	NEG
ND38	Y	Y	N	N	N/A	POS	POS	POS
ND39	Y	Y	N	N	N/A	POS	POS	NEG
ND40	Y	Y	N	N	N/A	POS	POS	N/A
ND41	Y	Y	Y	Y	N/A	GAD	NEG	N/A
UFM01	N	N	N	N	NEG	POS	NEG	NEG
UFM02	Y	Y	N	N	POS	NEG	NEG	NEG
UFM03	N	N	Y	Y	NEG	NEG	POS	NEG
UFM04	N	N	Y	Y	NEG	POS	POS	POS
UFM05	N	N	Y	Y	NEG	POS	NEG	NEG
UFM06	Y	Y	N	N	POS	NEG	NEG	NEG
UFM07	N	N	Y	Y	NEG	POS	NEG	NEG
UFM08	Y	Y	N	N	NEG	POS	NEG	NEG
UFM09	Y	Y	N	N	POS	NEG	NEG	NEG
UFM10	N	N	Y	Y	POS	NEG	NEG	NEG
UFM11	N	N	Y	Y	POS	POS	POS	POS
UFM12	N/A	N/A	N/A	N/A	NEG	POS	NEG	NEG
UFM13	N	N	Y	Y	NEG	POS	POS	POS

In addition, to examine the immune signature at the single cell level using transcriptomics, we obtained 130–160 ml of blood from two of the subjects with type 1 diabetes described above (two females, ages 30 and 34 years; duration of diabetes: 6 and 12 months) and identified as ND01 and ND02.

Ethical approval for this study was granted by the Ethics committee and institutional review board, and informed consent/assent was obtained from all subjects enrolled or their guardians.

### Detection of *β*-Cell-Specific CD4+ T Cells by Cytokine ELISPOT

Peptides representing native proinsulin epitopes (PIPs) or neoepitopes: hybrid insulin peptides (HIPs) (PIPs: C13–32; C19-A3; C22-A5, and HIPs: C peptide-IAPP1; C peptide-IAPP2; C peptide-NPY) were synthesized and purified by high-performance liquid chromatography (Thermo Hybaid, Germany).

Interferon-*γ* (IFN-*γ*), interleukin-17A (IL-17A), and IL-10 production by CD4+ T cells was detected by enzyme-linked immunospot (ELISPOT) assay, performed as described previously (2, 4), in triplicate for each peptide. Briefly, fresh PBMCs supplemented with peptide were dispensed into 48-well plates at a density of 3 × 10^6^ in 0.5 ml RPMI-1640 supplemented with antibiotics (TC medium; Life Technologies Ltd.) and 10% human AB serum (Sigma, Dorset, United Kingdom) and incubated at 37°C to a final concentration of 10 μg/ml. Control wells contained TC medium with an equivalent concentration of peptide diluent alone (DMSO). Pediacel, a penta-vaccine, was obtained from Sanofi Pasteur Ltd. (Guildford, U.K.) and used at 1 μl/ml to examine anamnestic responses induced by vaccination or infection as previously described (4).

Pre-warmed TC medium/10% AB serum was added 24 h later, and 48 h after stimulation, cells were washed and resuspended in TC medium containing 10% human AB serum and brought to a concentration of 10^6^/300 μl; 100 μl was dispensed in triplicate into wells of 96-well ELISA plates (Nunc Maxisorp; Merck Ltd., Poole, United Kingdom) pre-blocked with 1% BSA in PBS and precoated with monoclonal anti-IFN-*γ*, anti-IL-10, or anti-IL17 capture Ab (U-Cytech, Utrecht, The Netherlands). After capture at 37°C for 20–22 h, plates were washed in PBS/Tween 20, and spots developed according to the manufacturer’s instructions. Plates were dried, and spots of 80–120 μm were counted in a BioReader 3000 (BioSys, Karben, Germany), and data were expressed as stimulation index (SI) values (mean spot number of test peptide/mean spot number of diluent). The SI takes account of background, spontaneous responsiveness in the negative control, and ROC plots can be used to assign cut-off values as described previously; SI values ≥3 was taken to indicate a positive response for IFN-*γ* and IL-10 and ≥2 for IL-17A.

We have previously demonstrated that CD4+ T cells elicit the responses to the native peptides used in this assay by showing that depleting CD4+ cells abolished the cytokine responses (14).

### Isolation and Single Cell Transcriptional Profiling of *β*-Cell-Specific CD4+ T Cells

Peptides were pooled into two groups, those consisting of native epitopes in one and neoepitopes in the second group. The pools comprised of the three proinsulin peptides and included peptides of GAD (115–127 and 265–284) in the native group (pool 1) and deamidated and citrullinated versions of these in the neoepitopes pool [GAD 115–127 (120E) and GAD 265–284 (272cit.)] together with C-peptide IAPP1 and C-peptide-NPY peptides (pool 2); these were synthesized and purified by high-performance liquid chromatography (Thermo Hybaid, Germany). Each of the two experiments was conducted using blood obtained from two individuals with type 1 diabetes; PBMCs at 2 × 10^6^/ml were added to 20 wells of a 48-well plate; pool 1 peptides were added at a final concentration of 10 µg/ml to each well; this was repeated for an additional 20 wells where pool 2 was added at a final concentration of 10 µg/ml. Pediacel was used as positive control, and 1 μl/ml was added to a well containing cells at 2 × 10^6^/ml, and medium alone was used as a negative control. Anti-CD40 (Biolegend, clone G28.5, 303611) was added at a final concentration of 2 µg/ml to each well, and the cells were incubated for 18–20 h.

The cells were then harvested and washed with phosphate buffered saline (PBS). An antibody staining master mix consisting of anti-CD19 (M5E2, 561391), anti-CD3 (SK7, 641415), anti-CD4 (SK3, 345768), anti-CD45-RO (UCHL1, 337168), anti-CD95 (DX2, 561978), anti-CD154 (TRAP1, 555700), anti-CD69 (FN50, 555530) (BD Biosciences, Oxford, UK), and anti-CD27 [(O323, 302830) Biolegend, London UK] was prepared and spilt into five tubes. Anti-human Hashtag 1 (TotalSeqC 0251), anti-human Hashtag 2 (TotalSeqC 0252), anti-human Hashtag 3 (TotalSeqC 0253), anti-human Hashtag 4 (TotalSeqC 0254), and anti-human Hashtag 5 (TotalSeqC 0255) (Biolegend, London, UK) were added at a concentration of 0.1 µg/µl to each of the five tubes. The hashtags were used to barcode the samples as follows: patient 1 pool 1 (Hashtag 1); patient 1 pool 2 (Hashtag 2) patient 2 pool 1 (Hashtag 3); patient 2 pool 2 (Hashtag 4), and Pediacel (patients 1 and 2; Hashtag 5). The hashtag barcoded cells were incubated for 20 min in the dark and washed three times in FACS buffer (PBS with 3% FCS and 2% human AB) (both from Sigma, UK). CD4+ T cells responding to stimulation were identified by co-expression of CD69, and CD154 were sorted into 35 µl PBS using a BD FACSAria III cytometer as previously described (15).

Sorted cells were characterized using the 10× Chromium Controller Single-cell Immune Profiling system which simultaneously defines gene expression and T cell receptor profiles from single cells in the same sample; this was conducted at the Genomics facility at Guy’s and St Thomas’ Biomedical Research Centre. Briefly, single-cell suspensions were used to generate full-length cDNA in gel beads (containing Unique Molecular identifiers—UMI) in an emulsion master mix from 5′ V1 chemistry. The cDNA was amplified and subsequently size selected at the clean-up stage: smaller fragments were separated and stored for feature-barcoding/cell-hashing library preparation and the large fragments for gene expression (GEX) and T cell VDJ according to the manufacturer’s instructions.

The GEX libraries were enzymatically fragmented and indexed while the T cell library was first enriched [using Chromium Single Cell V(D) J Enrichment Kit, Human T Cell, PN-1000005] and then fragmented and indexed for further sequencing. All the three final indexed libraries (feature barcoded + GEX + VDJ) were diluted to 4 nM concentrations and then pooled together at a ratio of 1:4:1 (feature barcoded library: GEX library: VDJ library) before being sequenced at 10 pM on HisSeq Rapid flow cell using an Illumina’s HiSeq2500 instrument.

Cell ranger software (version 4) was used for calculating 5′ genes, and VDJ sequence construction and downstream secondary analysis were performed using Loupe Cell Browser (version 3.1.1.) and Seurat_3.2.2 in R (version 4.0.3).

Cell Ranger output was filtered to remove doublets, genes expressed in less than four cells, cells expressing less than 700 genes, and cells with more than 10% counts from mitochondrial gene transcription. We also eliminated all MAIT cells (identified by their TCR alpha TRAV1-2) and cells with double TCRB as potential doublets and cells identified as contaminating non-T cells in the first round of clustering. We then mapped all the remaining cells to a PBMC reference (16) (**Supplementary Figure 1**) with symphony and harmony and excluded all cells which, according to the outcome of knnPredict() function, were not CD4 T cells. We also assigned T cell subtypes whenever possible. Barcodes of cells used for the downstream analysis are included in **Supplementary File 1**.

For clustering purposes, each of 10× runs was split by individual and reintegrated with Seurat to remove individual effects.

Dimension reduction (PCA, UMAP) and clustering were performed on this cleaned dataset with exclusion of TCR, mitochondrial genes, and ribosomal protein genes. UMAP was performed on first 30 PCA axes and with 50 neighbors. Clusters were determined by SNN algorithm, with resolution of 0.2. Gene expression between clusters was compared with FindConservedMarkers() function in Seurat by MAST, and top genes (all with adjusted p values in each individual <0.0005 and fold change >2) are shown in **Figure 4C**. Comparison of gene expression between native and neoepitope pools was also performed in Seurat with MAST, with each individual included as a latent variable (results included in **Supplementary Table 1**).

Original Cell Ranger-reported clonotypes require all TCRB and TCRA chains of two cells to be identical to pertain to the same clone; we assumed that two cells are from the same TCR clone if they shared both TCR chains and there was no conflict in their B chains (so if they shared identical TCRA and TCRB, or two TCRA when at least one of the cells did not have TCRB reported). TCR sequences were compared using three-point matches (V-CDR3-J) and TCR chains within our experiments and against external databases when the data was available.

### Statistical Analysis

The proportion of individuals responding to individual peptides was compared using Fisher’s exact test and one-way ANOVA. Responses to native and neoepitopes were compared using paired t-tests. Differential gene expression between clusters and in response to peptide pools was tested with MAST method as implemented in Seurat, with individual as a latent variable. Frequency of clonotypes was determined by dividing the number of clonotypes by the total number of cells.

### Data Availability

A 10× data was submitted to Array Express (E-MTAB-10323).

## Results

### Comparison of T Cell Responses to Neoepitopes and Native Epitopes in Type 1 Diabetes and Preclinical Subjects by Cytokine ELISPOT

We set out to compare the magnitude, prevalence, and phenotype of the T cells responding to native and neoepitopes derived from islet autoantigens in individuals with or at risk of developing clinical type 1 diabetes using a sensitive cytokine ELISPOT. We selected native peptides of proinsulin (PIPs), representing naturally processed and presented epitopes known to elicit CD4 responses in PBMC from individuals with type 1 diabetes (2–4) and hybrid insulin peptides (HIPs), known to activate CD4 T cells isolated from pancreatic islets from deceased organ donors with type 1 diabetes (8, 9).

**Figure 1:**
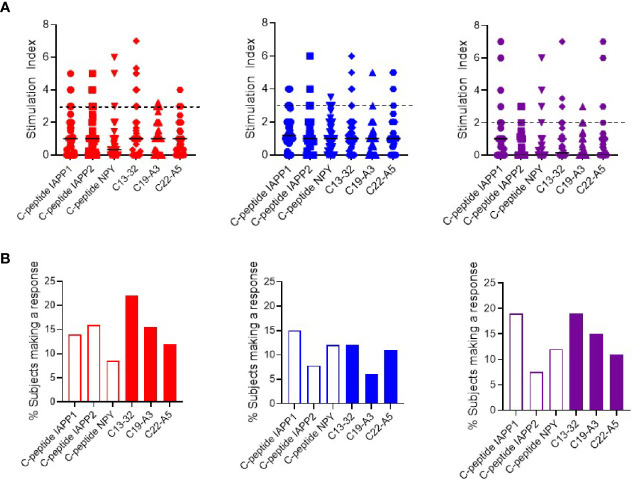
Cytokine T cell responses were measured by ELISPOT, and data are expressed as SI [stimulation index (mean spot number of test peptide/mean spot number of diluent)]. **(A)** Magnitude of IFN-*γ* (red) IL-10 (blue) and IL-17 (purple) responses in preclinical and subjects with type 1 diabetes against neo- (C-peptide-IAPP1, C-peptide-IAPP2 and C-peptide-NPY) and native epitopes (C13–32, C19-A3, C22-A5). The dashed line represents the cut-off for positivity for the stimulation index (three for interferon-γ and IL-10 and two for IL-17). **(B)** Prevalence of IFN-*γ* (red) IL-10 (blue) and IL-17 (purple) responses measured by ELISPOT in preclinical and subjects with type 1 diabetes against individual peptides of neo- (open bars) and native (filled bars) epitopes.

Firstly, we compared responses to native and neoepitopes in all individuals (pre-clinical and clinical type 1 diabetes). As depicted in **Figure 1A** and **Supplementary Figures 2A–C**, all peptides stimulated responses for all three cytokines in at least a proportion of individuals tested. All the peptides show a similar overall level of stimulation. Responses to Pediacel were detected in all of the patients for all three cytokines (**Supplementary Figures 3 **and** 4**).

As it is possible that the hierarchy of epitope immunodominance may be influenced by the stage of disease, we separated the subjects into patients with type 1 diabetes and those with preclinical disease. In subjects with type 1 diabetes, C-peptide-NPY elicits the highest interferon-*γ* response, C-peptide-IAPP2 the highest IL-10 and IL-17 responses were observed at a similar magnitude for C-peptide-IAPP1, C13-32, and C22-A5 (**Supplementary Figure 5A**). In preclinical subjects, C13–32 elicited the highest interferon-*γ* and IL-10 responses and C22-A5 the highest IL-17 response (**Supplementary Figure 6A**). However again, none of these differences reached statistical significance.

We have previously applied a ROC plot approach to establish criteria for defining a positive or negative response to individual peptide stimulation in cytokine ELISPOT assays (2, 4). We applied these criteria to the current data to determine the prevalence of positive responses in all the individuals tested and show that although there is a significant response to each peptide, there is no clear peptide immunodominance (**Figure 1B**). For interferon-*γ* responses, neoepitopes elicit a response in 8–14% of subjects and native epitopes in 12–22%. For IL-10, neoepitopes elicit a response in 8–15% of subjects and native epitopes in 6–12%, and finally IL-17 responses are present against 8–19% of neoepitopes and 11–19% of native epitopes.

Examining positive responses based on clinical group, in the subjects with type 1 diabetes, interferon-*γ* responses were observed in 6–16% against neoepitopes and 10–5% of native epitopes. IL-10 responses in 8–11% against neoepitopes and 0–12% of native epitopes and IL-17 against 10–26% of neoepitopes and 9–20% of native epitopes (**Supplementary Figure 5B**). In preclinical subjects, interferon-*γ* responses were observed against 8–23% of neoepitopes and 8–17% of native epitopes; IL-10 against 8–23% of neoepitopes and 8–31% of native epitopes, and no IL-17 responses were observed against neoepitopes and 8–15% subjects responded to native epitopes (**Supplementary Figure 6B**).

**Figure 2:**
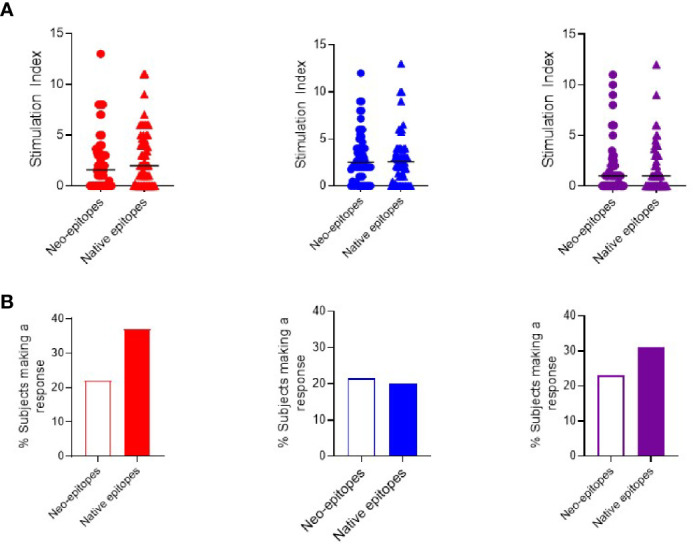
Cytokine T cell responses were measured by ELISPOT, and data are expressed as SI [stimulation index (mean spot number of test peptide/mean spot number of diluent)]. **(A)** Magnitude of responses as depicted by SI for IFN-*γ* (red) IL-10 (blue) and IL-17 (purple) responses against pooled neo- and native epitopes in preclinical and subjects with type 1 diabetes. **(B)** Prevalence of IFN-*γ* (red) IL-10 (blue) and IL-17 (purple) responses in preclinical and subjects with type 1 diabetes against pooled peptides of neo- (open bars) and native (filled bars) epitopes.

As responses to individual peptides were low, we pooled responses into two groups representing neo- or native epitopes and compared the magnitude of the response (**Figure 2A**) and the proportion of subjects mounting a positive response (**Figure 2B**). Although the overall magnitude of the response is similar, in terms of prevalence of positive responses, we observed a higher proportion of interferon-*γ* and IL-17 responses in response to native epitopes compared to neoepitopes (37 *vs.* 22% and 31 *vs.* 23% respectively). IL-10 responses were similar to both native and neoepitopes.

When segregating pooled responses by clinical group, the magnitude of response to neo- and native epitopes was similar in both groups (**Supplementary Figures 7A **and** 8A**). Examining the proportion of individuals with a positive response, we observed that subjects with type 1 diabetes trended towards higher interferon-*γ* responses to native *vs.* neoepitopes (45 *vs* 36% respectively). In contrast, in preclinical subjects, IL-10 and IL-17 responses were more prevalent to native epitopes (85 *vs* 62% (IL-10) and 31 *vs* 8% (IL-17) while interferon-*γ* responses were similar to both neo- and native epitopes (54%) (**Supplementary Figures 7B **and** 8B**).

Next, we compared the relative production of each cytokine stimulated by individual peptides to investigate if any single or group of epitopes led to a distinct polarization of the immune response.

**Figure 3:**
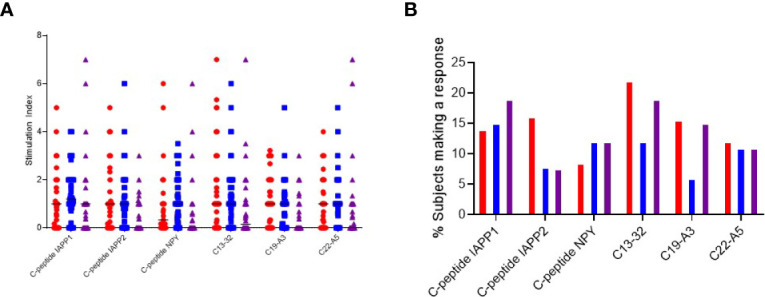
Cytokine T cell responses were measured by ELISPOT, and data are expressed as SI [stimulation index (mean spot number of test peptide/mean spot number of diluent)]. **(A)** Magnitude of IFN-γ (red) IL-10 (blue) and IL-17 (purple) responses depicted as stimulation indices for individual peptides of neoepitopes (three on the left) and native (three on the right) in preclinical and subjects with type 1 diabetes. **(B)** Frequency of IFN-*γ* (red) IL-10 (blue) and IL-17 (purple) responses to individual peptides of neoepitopes (three on the left) and native (three on the right) in preclinical and subjects with type 1 diabetes.

When examining the magnitude of response, each peptide was capable of stimulating a range of cytokines in different individuals with no peptide clearly eliciting a stronger response to a particular cytokine (**Figure 3A**). However, when examining the proportion of individuals who mount a positive response, we note that C-peptide-IAPP2 trends towards increased stimulation of a significant IFN-*γ* response, C13-32 and C19-A3 trend towards increased IFN-*γ* and IL-17 compared to IL-10, whereas the remainder showed no particular polarization (**Figure 3B**).

All the peptides except C19-A3 elicited multiple cytokines: interferon-*γ* and IL10 were detected in 3.8% of patients. C-pep-IAPP1 was the only peptide to elicit IL-10 and IL-17 responses, and these were detected in 3.8% of patients; interferon-*γ* and IL17 responses were only detected in one patient (1.9%), and in one patient, responses to all three cytokines were observed.

Finally, we examined responses to native and neoepitopes specifically in children as we have previously reported that native proinsulin epitopes are preferentially targeted by interferon-*γ* producing T cells in this group (4). We show that 11/14 (79%) of children made a response to the native epitopes compared to 8/14 (57%) to neoepitopes (p = 0.04). Furthermore, despite the small numbers in the current study, we confirm that as reported previously, the native epitope C13–32 elicits interferon-*γ* response in more than 40% of children with type 1 diabetes (**Supplementary Figure 9A**). Indeed, two thirds of all interferon-*γ* responses in all the subjects are detected in children. An interferon-*γ* response is also seen against the native epitope, C19-A3; in contrast, the neoepitopes tend to elicit IL-17 responses albeit at a lower frequency. IL-10 responses were seen in preclinical subjects particularly to C-pep-IAAP1; however, the number of subjects here are small (**Supplementary Figure 9B**).

Although the native peptides were initially identified as being HLA-DR4 restricted, we now know that these promiscuous responses are observed in non-HLA DR4 individuals, and for both native and neoepitopes we observed responses in non-HLA DR4/DQ8 individuals (**Supplementary Table 3**).

### Single Cell Transcriptional Profiling of CD4 T Cells Responding to Neo- and Native Epitopes

To further investigate the phenotype of neo- and native epitope-specific T cells, we conducted an unbiased transcriptional analysis including TCR clonotyping. Responding T cells were isolated based on CD154 and CD69 co-expression following brief *ex-vivo* culture with a pool of peptides representing native or neoepitopes and profiled using scRNA-seq *via* the 10× Genomics pipeline. As a positive control we used T cells activated by Pediacel from the same patients.

**Figure 4:**
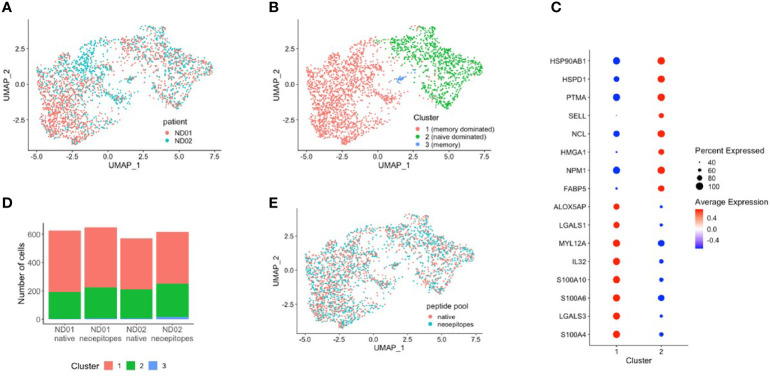
PBMCs from subjects ND01 and ND02 were stimulated with native (proinsulin peptides: C13–32, C19-A3, C22-A5; GAD peptides: 115–125, 265–284) and neoepitopes [C-pep-IAPP1, C-pep-NPY, GAD 115–127 (120E), GAD 265–284 (272 cit)] and each patient/pool was labeled with a different hashtag as described in *Methods*. Antigen-responsive cells were identified by expression of CD154/CD69, and immune profiling was subsequently conducted on single cells. Cells from subjects **(A)** ND01 and ND02 separate into **(B)** three clusters (0–3) based on gene expression similarity. **(C)** Clusters differ by expression of memory and naïve cell specific genes. **(D)** Cells reacting to native and neoepitopes are similarly split between clusters and **(E)** have similar patterns of expression.

Data from donors were filtered and integrated as described in *Methods*, and cells were projected in two dimensions (UMAP). Data for subjects ND01 and ND02 are shown in **Figure 4A**. We performed cell clustering based on gene expression and independently inferred cell subtype by mapping to a reference PBMC dataset (16). The main clusters corresponded to memory CD4 and naïve T cells (**Figure 4B**). In concordance with this, cluster markers are genes preferentially expressed in memory (S100A10, S100A4, LGALS3) or naïve (FABP, SELL) cells (**Figure 4C**; there is also a very small cluster—cluster 3 which comprises of memory-like cells with expression of PLZF.

Cells from both individuals are similarly split between clusters, although ND01 has higher fraction of Tregs and ND02 more naïve cells (**Supplementary Figures 10A, B**).

We then compared cells stimulated with peptide pools in each subject (**Figure 4D**) and show that for each subject, the composition of native and neoepitope stimulated samples does not differ in reference-based assignment or clusters.

Importantly, T cells responded to native or neoepitopes co-cluster (**Figure 4E**) for each individual, implying no general distinction between the responding cells.

We explored this further by comparing gene expression profiles of cells stimulated with either neo- or native epitopes for both patients and found no significant differences in gene expression (**Supplementary Table 1**).

**Figure 5:**
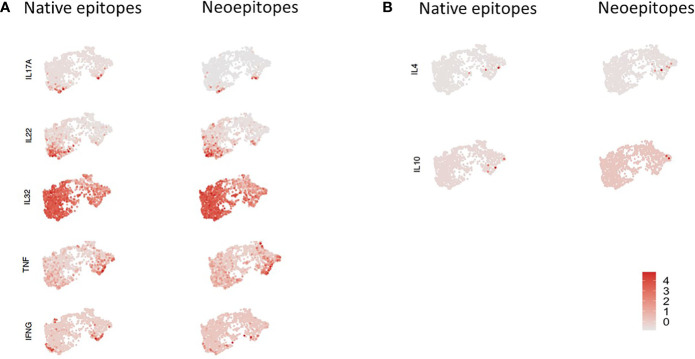
PBMCs from subjects ND01 and ND02 were stimulated with native [proinsulin peptides: C13–32, C19-A3, C22-A5; GAD peptides: 115–125, 265–284) and neoepitopes (C-pep-IAPP1, C-pep- NPY, GAD 115-127 (120E), GAD 265-284 (272 cit)]. Antigen-responsive cells were identified by expression of CD154/CD69, and immune profiling was conducted on single cells. **(A)** Cells were stimulated with native (right) and neoepitopes (left), and gene expression (depicted in pink) of **(A)** *IL-17A, IL-22, IL-32, TNF-α*, and *IFN-*γ and **(B)** *IL-4* and *IL-10* was examined. Level of expression corresponds to greater intensity of color.

We then examined expression of selected pro-inflammatory genes including *IL-17A, IL-22, TNF-α, INF-γ*, and *IL-32* and anti-inflammatory genes *IL-4* and *IL-10* in cells stimulated with either neo- or native epitopes (**Figure 5A**). *IL-17A* expression was generally low, although expression was slightly higher in patient ND01; *IL-22* expression was greater than *IL-17A*; similarly, expression of this was higher in patient ND01; there were no differences in cells stimulated with either neo- or native epitopes. *TNF-α* and *INF-γ* expression was higher in patient ND02, and expression in cells stimulated with either neo- or native epitopes was similar.

Interestingly, *IL-32* gene expression was by far the most extensive and present in both patients in response to both neo- and native epitope stimulated cells.

In contrast, expression of the anti-inflammatory cytokines (**Figure 5B**), *IL-4* and *IL-10* was undetectable in patient ND01 and very low in ND02 for both native and neoepitopes.

### TCR Use of Cells Responding to Neo- and Native Epitopes

The Single Cell Immune Profiling system allows the synchronized identification of gene expression and T cell receptor profiles from single cells in the same sample. We used this approach to assess the number and profile of TCR clonotypes and determine whether any are shared between T cells stimulated with either neo- or native epitopes.

**Figure 6:**
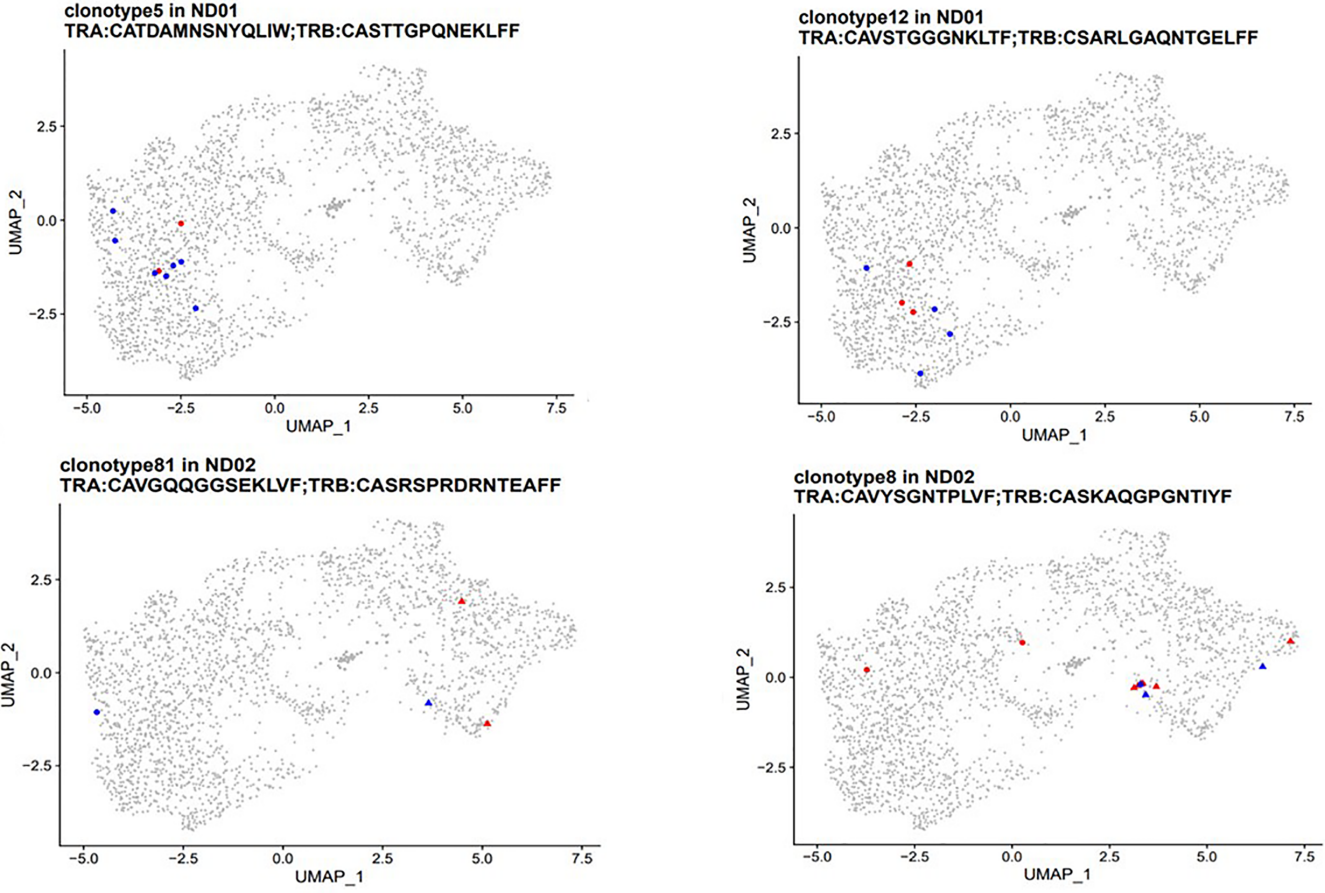
PBMCs from subjects with type 1 diabetes were stimulated with native [proinsulin peptides: C13–32, C19-A3, C22-A5; GAD peptides: 115–25, 265–284) and neoepitopes (C-pep-IAPP1, C-pep-NPY, GAD 115–127 (120E), GAD 265–284 (272 cit)]. Antigen-responsive cells were identified by and sorted on expression of CD154/CD69. Immune profiling was conducted on single cells from which cDNA was synthesized, followed by library construction and sequencing as described in *Methods*. TCR clonotypes (both *α* and *β* chains) were examined in patients ND01 (top panel) and ND02 (bottom panel). T cells responding to native epitopes are shown in red and those responding to neoepitopes are shown in blue. Clusters are marked by point shapes (circle, triangle, and square).

For patient set ND01/ND02 we identified 76 clonotypes shared between T cells responding to native and neoepitopes in subject ND01 and seven such clonotypes for ND02 (**Table 2**). Each of these TCR receptors was present in 2–30 cells, and again there was no preference towards either native or neoepitopes. Of the 76 clonotypes in ND01, four of the TCRB sequences (highlighted in red) corresponded to sequences in the JDRF npod database (http://clonesearch.jdrfnpod.org) where they were present in the pancreatic lymph nodes and spleen of both patients with type 1 diabetes and healthy controls (17) (**Table 2**). Representative data are shown in **Figure 6**.

**Table 2:** T cell clonotypes shared amongst native and neoepitopes.

Patient Set & Clonotype	Subject	TRA	TRB	Cells’ native epitopes	Cells Neo-epitopes
ND01/ND02 1	ND01	CALISGGGADGLTF	CSVGSQPQHF	21	11
ND01/ND02 4	ND01	CAAINAGNNRKLIW	CAWNKKKGLTYNEQFF	5	5
ND01/ND02 5	ND01	CATDAMNSNYQLIW	CASTTGPQNEKLFF	2	7
ND01/ND02 9	ND01	CAVGTGTASKLTF	CASSTTSLSSYEQYF	4	2
ND01/ND02 12	ND01	CAVSTGGGNKLTF	CSARLGAQNTGELFF	4	3
ND01/ND02 13	ND01	CAVPRSGNTGKLIF	CASSLPGVTNYGYTF	3	4
ND01/ND02 20	ND01	CAANMYALNF	CASSPGQGANTGELFF	4	2
ND01/ND02 21	ND01	CAGIQGAQKLVF	CASSFIAGGPSDTQYF	1	1
ND01/ND02 27	ND01	CILRDSGATNKLIF	CAAGQPNTGELFF	3	3
ND01/ND02 273	ND01	CAVPDQTGANNLFF	CASSTHRVNAEAFF	1	1
ND01/ND02 276	ND01	CALMNQGGKLIF	CSVEDPDSRTDTQYF	1	1
ND01/ND02 19	ND01	CAVISNTGKLIF	CASSRGQGAEKLFF	3	2
ND01/ND02 23	ND01	CATDPWSGANSKLTF	CASGFTGGYNEQFF	3	1
ND01/ND02 230	ND01	CASSGGSYIPTF	CASSLGRGASTEAFF	1	1
ND01/ND02 234	ND01	CVARRGGGGNKLTF	CASSPGTGQETQYF	1	1
ND01/ND02 2351/3227	ND01	CAVRSNQAGTALIF CAVGGNTNAGKSTF CAVRSNQAGTALIF	CASSAAGASSYEQYF CASSAAGASSYEQYF	1	1
ND01/ND02 28	ND01	CLVGDISTSGTYKYIF	CASRHARQPQHF	3	2
ND01/ND02 287	ND01	CAGSGTMNYGGSQGNLIF	CASSGQGGGNTEAFF	1	1
ND01/ND02 30	ND01	CVVTGYNNNDMRF	CAIREGYGYTF	2	3
ND01/ND02 37	ND01	CAGPDMDSNYQLIW	CASRYRGGSGRELFF	2	3
ND01/ND02 38	ND01	CAFIWGSSNTGKLIF	CASRRGQANYGYTF	2	1
ND01/ND02 48	ND01	CILARSGAGSYQLTF	CASSYFGRGTDTQYF	3	2
ND01/ND02 18	ND01	CVVNTVTGGGNKLTF	CASSLRGPYGYTF	2	2
ND01/ND02 22	ND01	CAVRIQGAQKLVF	CASSYSEVYNEQFF	2	2
ND01/ND02 26	ND01	CALSASKIIF	CASSLSRESNQPQHF	1	1
ND01/ND02 263	ND01	CALSDRPGSARQLTF	CSASPTPQVGGTEAFF	1	1
ND01/ND02 264	ND01	CALPLSKTGANNLFF	CASSSTGGYEQYF	1	1
ND01/ND02 221	ND01	CALRSGANSKLTF	CASSLSLAPSDEQFF	1	1
ND01/ND02 2297/6	ND01	CAVSDTGGFKTIF CAVGPAGTGGFKTIF CAVSDTGGFKTIF	CATSETGKYQETQYF CATSETGKYQETQYF	4	5
ND01/ND02 29	ND01	CAVNPGNTPLVF	CSARDDRTAAEAFF	3	1
ND01/ND02 31/42	ND01	CAVADSNYQLIW CAVSYTGGGNKLTF CAVSYTGGGNKLTF	CASSSLTGGLYNEQFF CASSSLTGGLYNEQFF	4	5
ND01/ND02 32	ND01	CAVRDGGTGGFKTIF	CASSLGAQYTGELFF	1	1
ND01/ND02 33	ND01	CAAPLKTSYDKVIF	CASSLDRGVQPQHF	2	2
ND01/ND02 335	ND01	CAVEDGGNDYKLSF CAVVPPFTGGGNKLTF	CASSAGTGRHTDTQYF	1	1
ND01/ND02 349	ND01	CALSEARDAGNMLTF	CSARGQGVATSNQPQHF	1	1
ND01/ND02 319	ND01	CLVGDIRGGGYQKVTF	CSVEVDRAGTEAFF	1	1
ND01/ND02 43	ND01	CAASGWGSARQLTF	CASSYRAPGDNSPLHF	2	2
ND01/ND02 47	ND01	CALKAAGNKLTF	CASAGEHTGELFF	3	1
ND01/ND02 49	ND01	CAPRGNDYKLSF	CSAIDNTDTQYF	2	2
ND01/ND02 53	ND01	CAVDAAGNKLTF	CASSADILLREQYF	3	1
ND01/ND02 54	ND01	CAAGGATNKLIF	CASSPRALENTEAFF	1	1
ND01/ND02 57	ND01	CAVSNQAGTALIF	CASSLRGTGGYTF	1	1
ND01/ND02 58	ND01	CALSENTNAGKSTF	CSAQGGGDTEAFF	1	2
ND01/ND02 62	ND01	CALSRTGYSTLTF	CASVVLGNTEAFF	3	1
ND01/ND02 69	ND01	CAVFTGGFKTIF	CATSVRGDYNEQFF	2	2
ND01/ND02 11	ND01	CAVYHAGNMLTFT	CASSTGTGGYEQYF	1	2
ND01/ND02 113	ND01	CAGAGGSYIPTF	CASSPGGPVGNTIYF	1	1
ND01/ND02 116	ND01	CAMRKGDYKLSF CAVLGGGGFKTIF	CASSAGRGSDYGYTF	1	1
ND01/ND02 117	ND01	CAASAIKYGGSQGNLIF	CASSQDTASGAYEQYF	1	1
ND01/ND02 140	ND01	CAASAALQTGANNLFF	CASSLGQGAEAFF	2	1
ND01/ND02 142	ND01	CALTSRGGFGNVLHC	CSAREGAGANVLTF	2	1
ND01/ND02 151	ND01	CAVKEVDSSYKLIF	CASSTGTGAEMNTEAFF	1	1
ND01/ND02 153	ND01	CARGNNDYKLSF	CASSATLQGGGYTF	1	1
ND01/ND02 1584/52	ND01	CILRDAFGNEKLTF CILRDAFGNEKLTF CAGHNNAGNMLTF	CSARRDLGNQPQHF CSARRDLGNQPQHF	2	1
ND01/ND02 247	ND01	CAAMNTGGFKTIF	CASSELSSGRNNEQFF	1	1
ND01/ND02 248	ND01	CAVGAGYGGATNKLIF	CASSRGVTEAFF	1	1
ND01/ND02 257	ND01	CALMNTGFQKLVF CSLRYSGAGSYQLTF	CASSFGLRQGGRVGEEYF	1	1
ND01/ND02 1878/44	ND01	CASQRGSQGNLIF CALNTGTASKLTF CALNTGTASKLTF	CASSSGLAGGLEQYF CASSSGLAGGLEQYF	1	1
ND01/ND02 190	ND01	CALRDTGGFKTIF	CASSAGTGGLFGELFF	1	1
ND01/ND02 199	ND01	CAVASNTGKLIF	CSVEDSGNTIYF	1	1
ND01/ND02 205	ND01	CALSSQGTYKYIF CAGVFMRF	CASSETGRGIEQYF	1	1
ND01/ND02 207	ND01	CATHASGGSYIPTF	CASKNQGTYGYTF	1	1
ND01/ND02 209	ND01	CAVGIAGNTPLVF	CASSPSWDFHGYTF	1	1
ND01/ND02 64	ND01	CAVPNQAGTALIF	CASSQRGTYEQYF	2	1
ND01/ND02 65	ND01	CATDTSYTGANSKLTF	CASSRILTSGNRGVTQYF	2	1
ND01/ND02 67	ND01	CAVGVSGNTPLVF	CASSVLSGGETQYF	1	2
ND01/ND02 75	ND01	CVVSDRDTGFQKLVF	CASSVAGSVSDTQYF	2	1
ND01/ND02 77	ND01	CALISGGGADGLTF		1	1
ND01/ND02 78	ND01	CVVGDFGNEKLTF	CASSLVQAYYSGNTIYF	2	1
ND01/ND02 79	ND01	CALSGGGSARQLTF	CASSSSLRRFNYGYTF	2	1
ND01/ND02 82	ND01	CAFRGTGNQFYF	CASSITGTTYEQYF	1	2
ND01/ND02 83	ND01	CAGRSGGYQKVTF	CSVERRGGDTQYF	1	2
ND01/ND02 91	ND01	CAVIPMNSGYSTLTF	CASSPRPQGQSYNEQFF	2	1
ND01/ND02 92	ND01	CAVGPWGNEKLTF	CASSLDSKPEAFF	2	1
ND01/ND02 94	ND01	CAASLGAGSYQLTF	CSARGAGGLTYEQYF	1	2
ND01/ND02 98	ND01	CAASIRSGTYKYIF	CASSLRLTGGNNEQFF	2	1
ND01/ND02 99	ND01	CAANDQTGANNLFF	CASRKAGGPYEQYF	1	2
ND01/ND02	ND02	CASSPQGGSEKLVF	CASSFFRGRNTEAFF	1	2
ND01/ND02 8	ND02	CAVYSGNTPLVF	CASKAQGPGNTIYF	3	7
ND01/ND02 81	ND02	CAVGQQGGSEKLVF	CASRSPRDRNTEAFF	2	2
ND01/ND02 194	ND02	CAASRDTGFQKLVF	CASSRTGRTDQPQHF	1	1
ND01/ND02 196	ND02	CAMREGPNDYKLSF	CSARDFLRLAGGEETQYF	1	1
ND01/ND02 200	ND02	CAVGTQGGSEKLVF	CASRSNRDRNTEAFF	1	1
ND01/ND02 362	ND02	CIVGYSTLTF	CASSLARLVAGGDNEQFF	1	1

Sequences highlighted in red correspond to those listed in the JDRF npod TCR database.

For both sets of patients, stimulating T cell with native or neoepitopes did not lead to a preferential expansion of specific clonotypes shared between peptide pools.

Finally, we also investigated TCR clones that were expanded by one peptide pool only (**Supplementary Table 2**). Subject ND01 had identical number of clonotypes, with similar number of cells expanded to either native or neoepitope pools. In contrast, ND02 had more expanded clones in the native epitope pool compared to the neoepitope pool (eight *versus* one). Eight of the TCRB chains were also present in the JDRF npod database, where they were present in the pancreatic lymph nodes and spleen of both patients with type 1 diabetes and healthy controls at frequencies ranging from 0.0008 to 0.002% which is comparable to the 0.004–0.005% for both native and neoepitopes in the present study. Taken together, these data show the need for testing more patients to determine whether there is a quantitative difference in expanded clonotypes between native and neoepitopes.

## Discussion

In agreement with observations made by others we can detect T cell responsive to HIPs in PBMCs and that these cells respond with a range of cytokine production. We wanted to determine if they were present at a higher frequency or with a different phenotype to PIPS and demonstrated that this is not the case. Our findings contrast with those recently published by Mitchell et al. (18); however, we did not test all the HIPs that they used. It is possible that some HIPs elicit stronger responses than those we tested, but equally some PIPs may elicit stronger responses than the ones they tested—there was only one PIP in their panel and that differed in amino acid sequence to the three used in our panel. Moreover, in that study there was no difference between the negative control (no antigen) and B9-23 in either autoantibody-positive or autoantibody-negative individuals for either IFN-*γ* or IL-10; for C10-24, they report very low reactivity too indicating that overall, they saw very low reactivity to native peptides in their study.

In the present study, through a detailed phenotypic analysis, we show that HIPs are not better than conventional islet epitopes in eliciting IFN-*γ*, IL-10, or IL-17 specific T cell responses in both patients with type 1 diabetes and high-risk unaffected family members. On the contrary, pro-inflammatory responses tend to be more frequent to islet epitopes when responses are measured collectively. Furthermore, in terms of epitope hierarchy, the native epitope, C13-32, elicits the most frequent IFN-*γ* and IL-10 responses in patients with type 1 diabetes and preclinical subjects respectively consistent with our previous studies (3, 4). This was confirmed in a subgroup analysis on children with type 1 diabetes where interferon-*γ* responses to all the PIPs including C13–32 were higher than those observed against HIPS despite comparable HLA-DR4 and HLA-DQ8 prevalence.

The three HIPs used here were selected based on two previous studies which described IFN-*γ* producing CD4 T cell clones specific for these HIPS isolated from islets of type 1 diabetes patients (8, 9).

During the course of this study, Baker et al., published data measuring IFN-*γ* T cell responses by ELISPOT against a series of HIPs including the three used in the present study and showed minimal reactivity: C-peptide-IAAP1 elicited IFN-*γ* responses in 0/35 patients and both C-peptide-IAAP2 and C-peptide NPY in 1/35 patients each (3%) (11). In contrast, we detected reactivity in 16% of subjects with type 1 diabetes for C-peptide-IAAP1; reactivities to C-peptide-IAAP2 and C-peptide NPY were also higher than those reported by Baker et al. (14% and 6% respectively). We also show frequent IL-17 T cell responses to C-peptide-IAAP1 which suggests that this epitope may be relevant in type 1 diabetes as it elicits multiple cytokine reactivity.

Baker et al. also tested responses against the native Ins75–90 peptide and report reactivity in ~10% of patients; this peptide corresponds to Ins75–92 (C19-A3) which we have used not only in the present study but extensively in the past and reported reactivity in more than 50% of subjects with type 1 diabetes (4). Due to limitations in the volume of blood available, it was beyond the scope of this study to test a wider selection of peptides presented by overlapping HLAs, but the frequencies of DR4 and DQ8 were compatible (63% and 56% respectively). Furthermore, using a single peptide concentration for stimulation does not allow us to differentiate between higher and lower affinities of the native and neoepitopes for MHC.

These variations in frequency of responses can be attributed to differences in assay design; Baker et al. stimulated their cells for 96 h prior to transfer to anti-IFN-*γ* antibody coated plates whereas we stimulate for 48 h, and they also report a much higher background response which could be due to differences in the serum/media used, during cell transfer to the anti-IFN-*γ* coated plate and the longer incubation time. The fact that cytokine responses to Pediacel were detected in all the subjects does validate the robustness of the ELISPOT assay used in the current study.

The lack of phenotypic differences in T cells responding to either native or neoepitopes is further substantiated by the Single Cell Immune-Profiling data which detects subtle differences in gene expression. Intriguingly, when focusing on pro-inflammatory cytokines, we noted that IL-32 expression was very high in patients responding to both native and neoepitopes. This ubiquitous expression could be because of culture conditions, but it is unlikely as its expression is dependent on stimulation (19). A potential role for IL-32 in type 1 diabetes has been suggested in two recent studies describing gene upregulation in pancreatic *β* cells (20) and the detection of *IL32* transcripts preceding the onset of autoantibodies in type 1 diabetes (21). IL-32 has been implicated in inflammatory bowel disease (IBD) and rheumatoid arthritis (RA) (22) where it is thought to orchestrate a panoply of other cytokines; indeed, in RA, IL-32 participates in the interplay with IL-17 in disease pathogenesis by amplifying inflammation in the synovium (23). Based on these data and previous studies on the role of IL-17 in T1D (2, 24), the elevated expression of IL-32 described in the present study warrants further investigation, and future studies will address whether IL-32 is produced by T cell stimulated with islet autoantigenic peptides. Overall, the lack of differences in gene expression and the similarity in composition of cells within clusters further substantiate that there is no difference in T cells responding to native or neoepitopes.

Finally, we were able to assess the number and profile of clonotypes present in immune response upon stimulation. Further evidence of the lack of distinction between T cells responding to native compared to neoepitopes comes from the T cell clonotype data which shows no preferential expansion of clonotypes. For this part of the study the T cells were stimulated with native peptide pools consisting of proinsulin and GAD or neoepitope peptide pools consisting of HIPs and modified GAD epitopes. There is very little sequence homology between the proinsulin and hybrid insulin peptides in the native and neoepitopes pools, whereas there is only one amino acid difference between the two GAD epitopes (deamidation and citrullination) in the two pools thus implying that the shared clonotypes must be targeting one of the GAD peptides. This is supported by the fact that clonotype 1 in patient ND03 has the TCRB chain sequence: CASSPATGGSSYNEQFF which has been previously identified in T cells stimulated with GAD (25).

Interestingly, four of the shared clonotypes in the current study have TCRB sequences found in the nPOD TCR database where they are present in the pancreatic lymph nodes and spleens of patients with type 1 diabetes. The shared TCR clonotypes between cells stimulated by the native and neoepitope pools suggest a lineage relationship between cells recognizing the two types of epitopes, although the specificity of these clonotypes has not been demonstrated. TCRs unique to either native or neoepitopes were also detected, but the specificity is not known. Future work will address the exact specificity of the epitope by transducing the TCR sequences into immortalized TCR−/− cell lines such as Jurkat cells and screening the relevant peptides.

We were only able to test two patients in the single Cell Immune profiling system, and our peptide panel for both native and neoepitopes had to be limited as we were constrained in the volume of blood available especially from juvenile subjects—this is a potential weakness in the study; however, the Immune Profiling data can be regarded as exploratory and can be used to guide future studies.

In summary, neoepitopes in T1D have a significant role in type 1 diabetes pathology; however, as the list of potential targets grows there is an even more pressing need to appraise and validate these epitopes and determine whether any have biomarker potential. By using a combination of phenotypic, transcriptomic, and clonotypic analyses we show that as it stands, neoepitopes are comparable to the native epitopes currently in use for immuno-monitoring studies.

## Data Availability Statement

The raw data supporting the conclusions of this article will be made available by the authors, without undue reservation.

## Ethics Statement

The studies involving human participants were reviewed and approved by the National Research Ethics Committee, Bromley NRES Committee, reference number 08/H0805/14. Written informed consent to participate in this study was provided by the participant or the participants’ legal guardian/next of kin.

## Author Contributions

SA designed and performed experiments, analyzed data, and wrote the manuscript. IP-A designed experiments and analyzed the data, YK, EW, NY, CD-V, YS, EP, and LK performed experiments and analyzed data. AL analyzed data and reviewed the manuscript. SA and MP designed research studies. SA and MP conceived ideas and reviewed the manuscript. SA and MP oversaw research. TT reviewed and edited the manuscript. MP is the guarantor of this work and, as such, had full access to all the data in the study and takes responsibility for the integrity of the data and the accuracy of the data analysis. All authors contributed to the article and approved the submitted version.

## Funding

This project has received funding from JDRF Research grant: 2-SRA-2017-510-S-B and the Innovative Medicines Initiative 2 Joint Undertaking under grant agreement No 115797 (INNODIA) and No 945268 (INNODIA HARVEST). This Joint Undertaking receives support from the Union’s Horizon 2020 research and innovation programme, “EFPIA”, “JDRF”, and “The Leona and Harry B. Helmsley Charitable Trust”.

## Conflict of Interest

The authors declare that the research was conducted in the absence of any commercial or financial relationships that could be construed as a potential conflict of interest.
