# Supplementary material for: Mapping T Cell Responses to Native and Neo-Islet Antigen Epitopes in at Risk and Type 1 Diabetes Subjects
Source: Front Immunol. 2021 Jun 25;12:675746. doi: 10.3389/fimmu.2021.675746 (PMC8274489; doi:10.3389/fimmu.2021.675746)
Supplement: Supplementary file 2 [file DataSheet_2.pdf]

Supplementary Figure 1

a

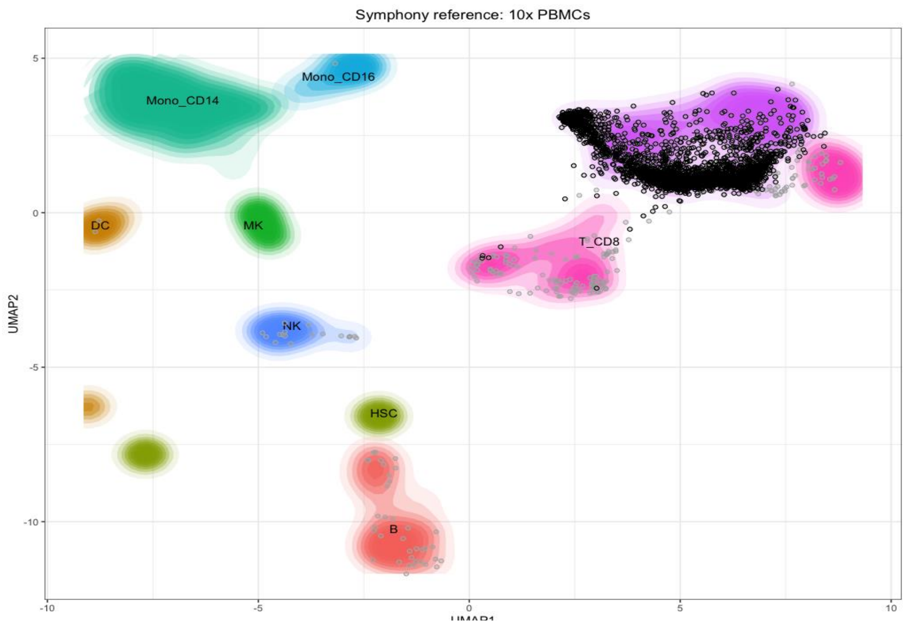

b

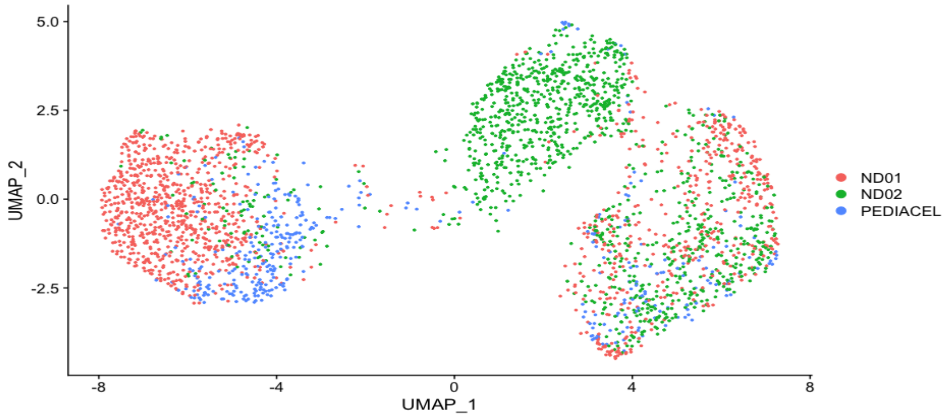

(a) Mapping of cells from both experiments to a PBMC reference set. Cells marked in black were analysed downstream, in grey filtered out cells.

(b) Filtered data from experiment using samples ND01 and ND02. Cells cluster to large extent by individual, suggesting integration removing individual effect is necessary

Supplementary Figure 2

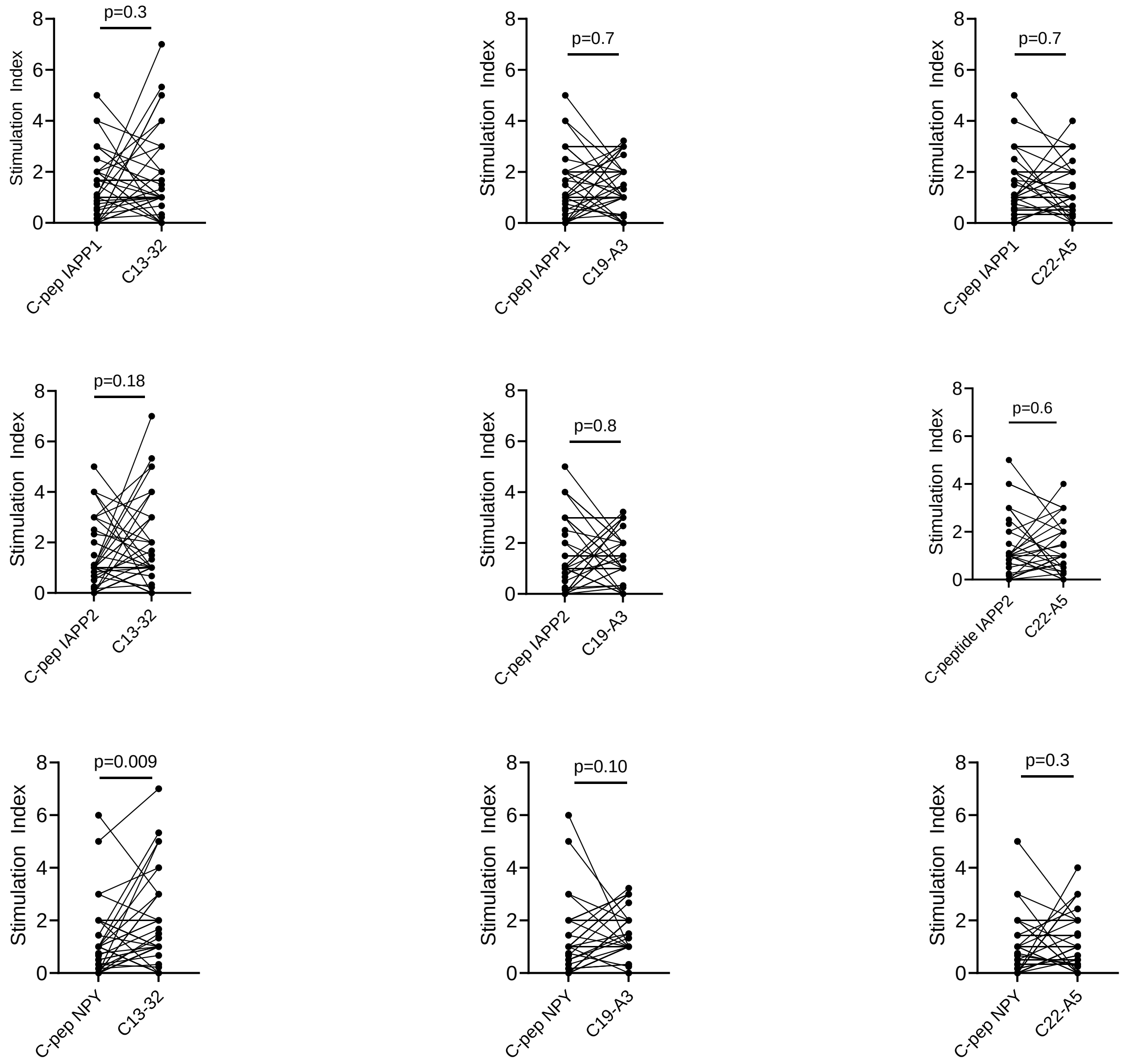

Supp. Figure 2(a): Graphs comparing IFN-γ ELISPOT responses between neo- and native epitopes with lines connecting paired samples from individuals.

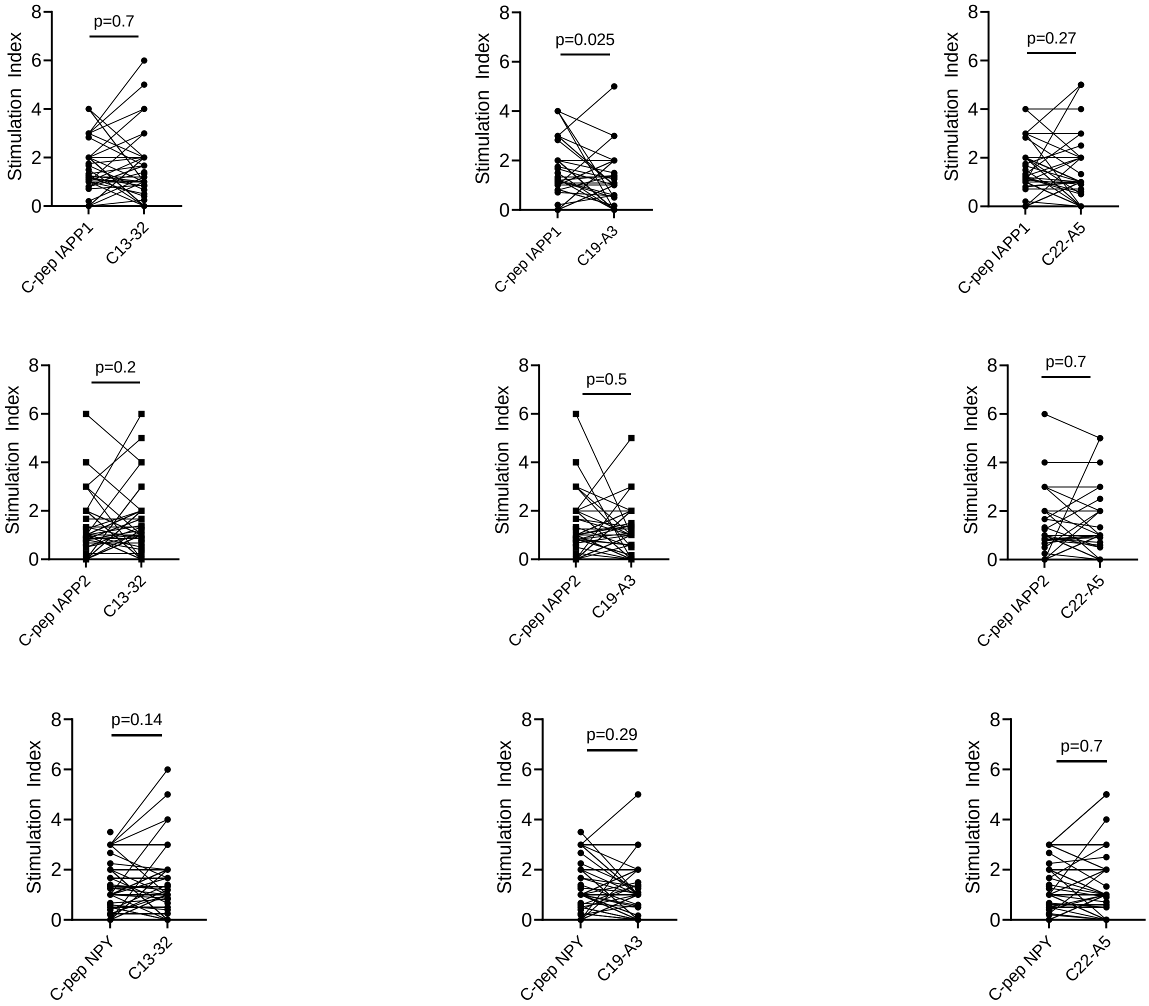

Supp. Figure 2(b): Graphs comparing IL-10 ELISPOT responses between neo- and native epitopes with lines connecting paired samples from individuals.

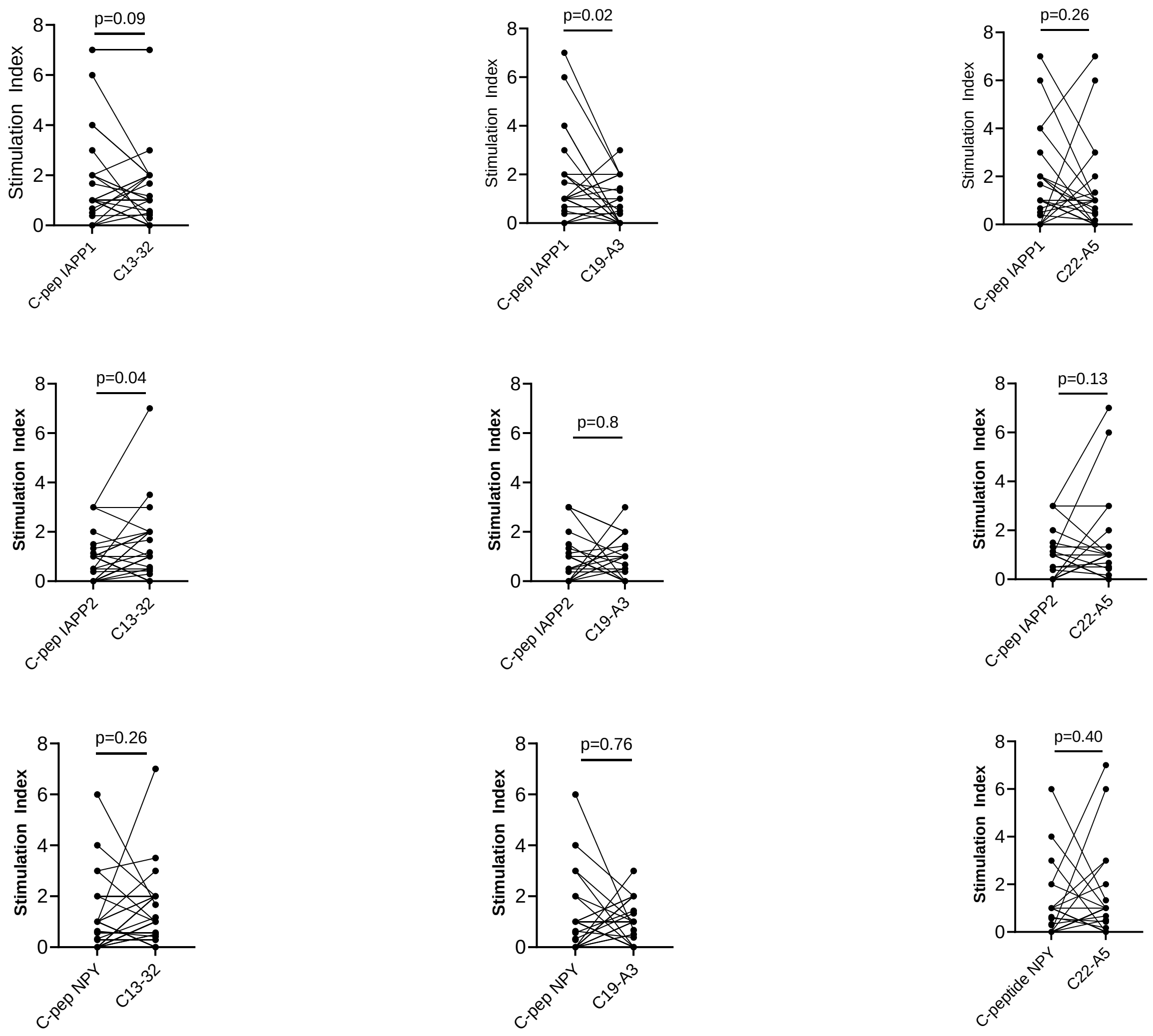

Supp. Figure 2(c): Graphs comparing IL-17 ELISPOT responses between neo- and native epitopes with lines connecting paired samples from individuals.

Supplementary Figure 3

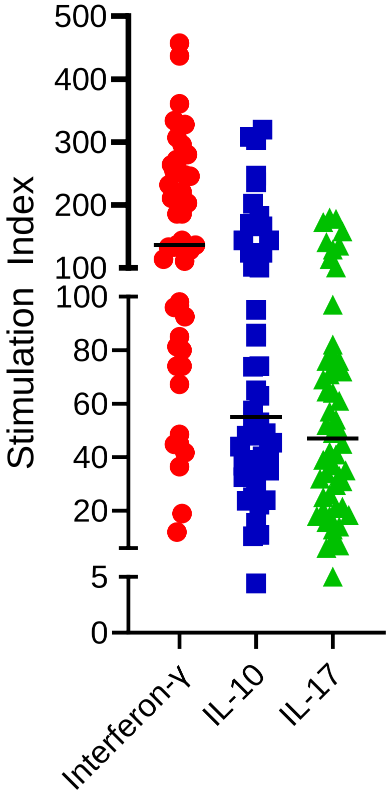

Supp. Figure 3: Magnitude of responses to Pediacel as depicted by SI for IFN-γ (left) IL-10 (centre) and IL17 (right) for all subjects. Positive response to Pediacel are detected in all subjects for all 3 cytokines.

Supplementary Figure 4

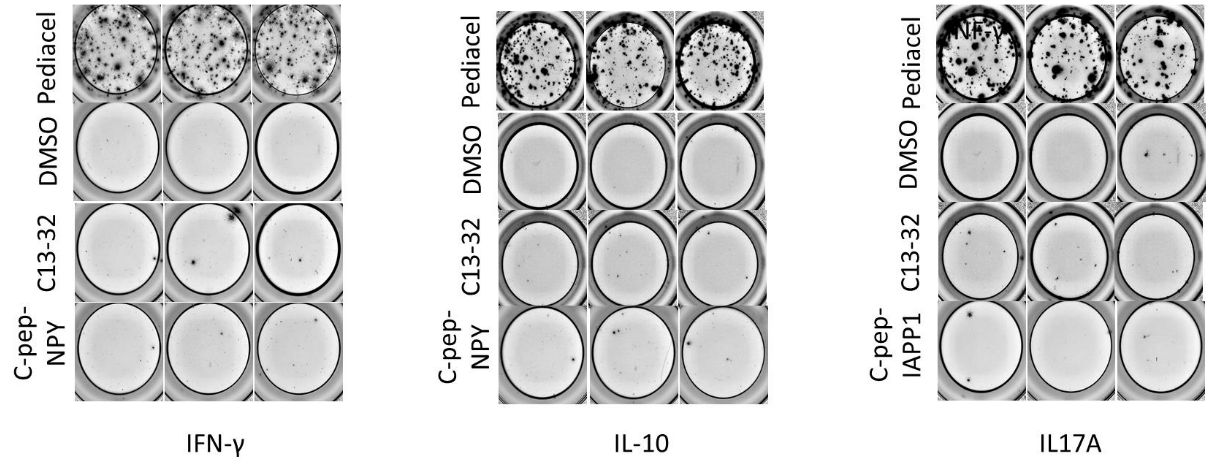

Supp. Figure 4: Examples of IFN-γ (left), IL-10 (centre) and IL17 (right) T cell responses to Pediacel (top panel), the peptide diluent, DMSO (second row) and neo- and native epitopes (bottom 2 panels).

Supplementary Figure 5

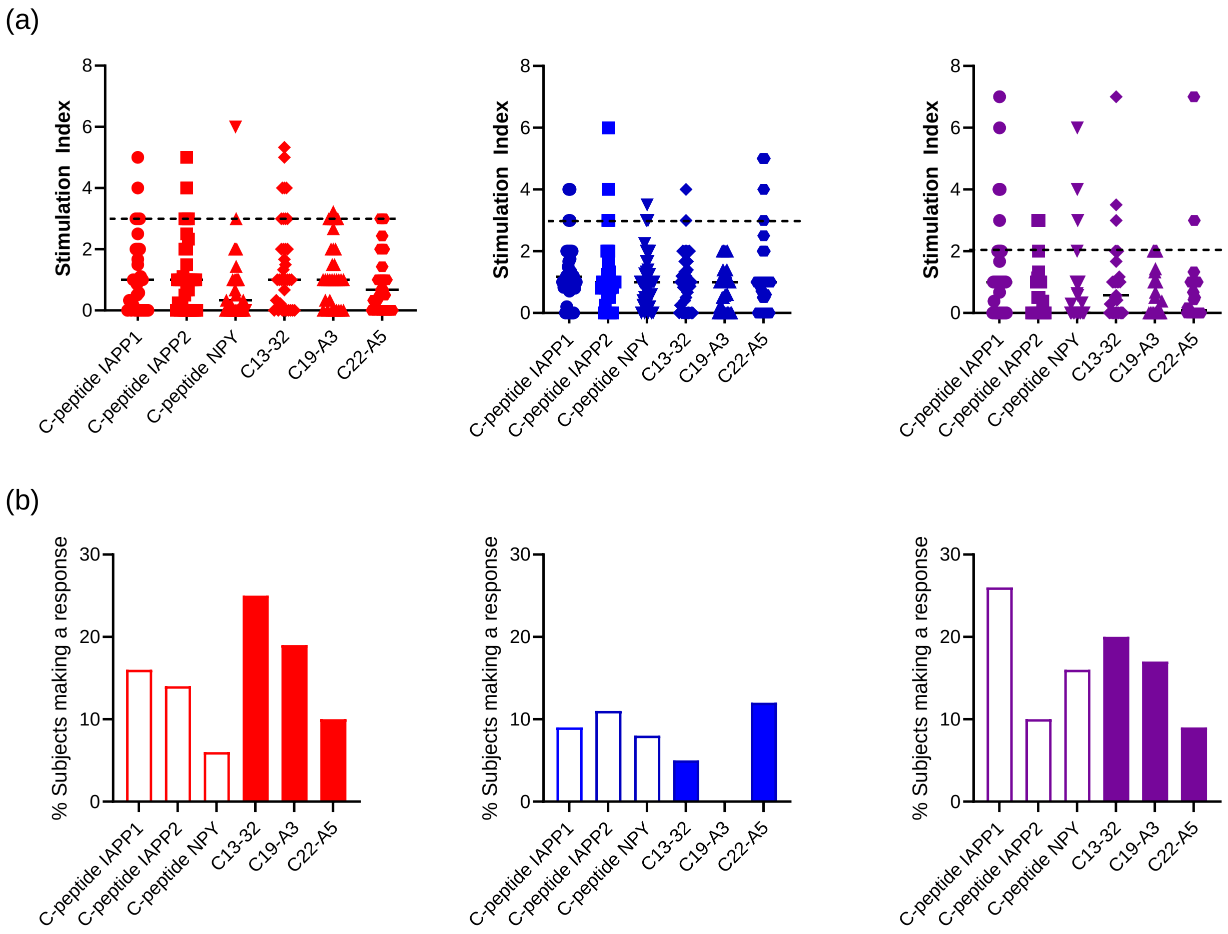

Supp. Figure 5(a): Magnitude of IFN-γ (left) IL-10 (centre) and IL-17 (right) responses in subjects with type 1 diabetes against neo- (C-peptide-IAPP1, C-peptide-IAPP2 and C-peptide-NPY) and native epitopes (C13-32, C19-A3, C22-A5). The dashed line represents the cut-off for positivity for the stimulation index.

Supp. Figure 5(b): Prevalence of IFN-γ (left), IL-10 (centre) and IL-17 (right) responses in subjects with type 1 diabetes against individual peptides of neo- (open bars) and native (filled bars) epitopes.

Supplementary Figure 6

(a)

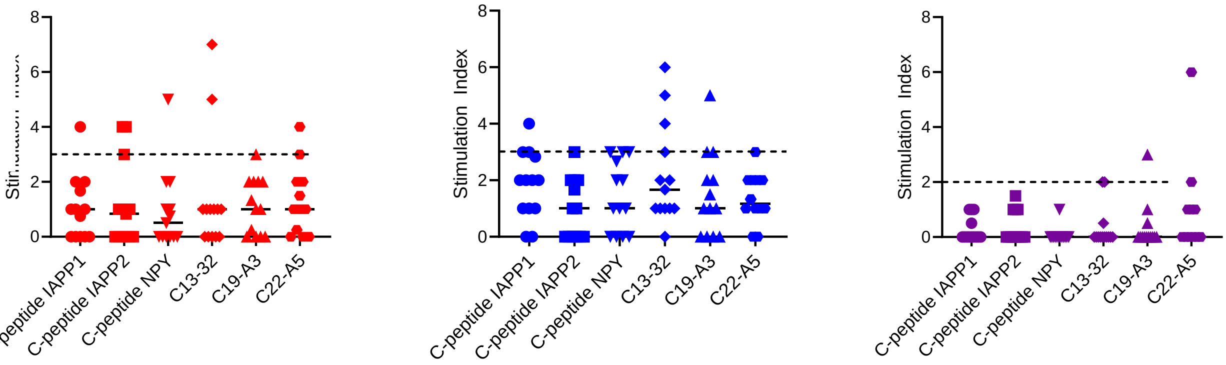

(b)

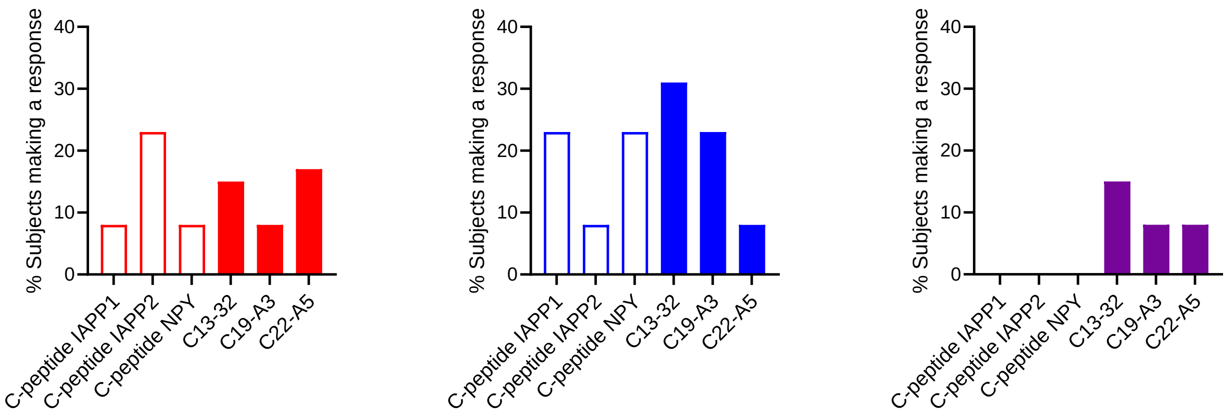

Supp. Figure 6(a): Magnitude of IFN-γ (left) IL-10 (centre) and IL-17 (right) responses in preclinical subjects against neo- (C-peptide-IAPP1, C-peptide-IAPP2 and C-peptide-NPY) and native epitopes (C13-32, C19-A3, C22-A5). The dashed line represents the cut-off for positivity for the stimulation index.

Supp. Figure 6(b): Prevalence of IFN-γ (left), IL-10 (centre) and IL-17 (right) responses in preclinical subjects against individual peptides of neo- (open bars) and native (filled bars) epitopes.

Supplementary Figure 7

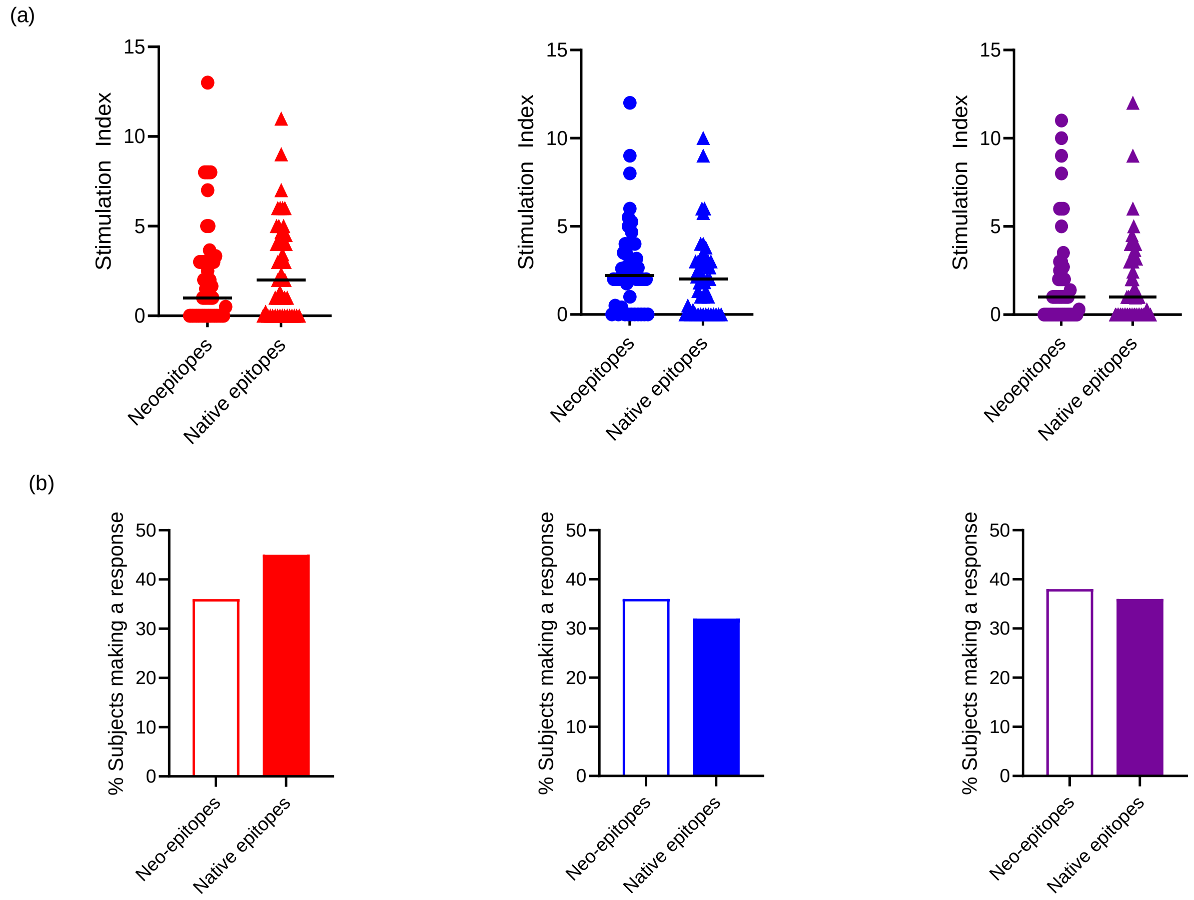

Supp. Figure 7(a): Magnitude of responses as depicted by SI for IFN-γ (left) IL-10 (centre) and IL17 (right) responses against pooled neo- and native epitopes in subjects with type 1 diabetes.

Supp. Figure 7(b): Prevalence of IFN-γ (left), IL-10 (centre) and IL-17 (right) responses in subjects with type 1 diabetes against pooled peptides of neo- (open bars) and native (filled bars) epitopes.

Supplementary Figure 8

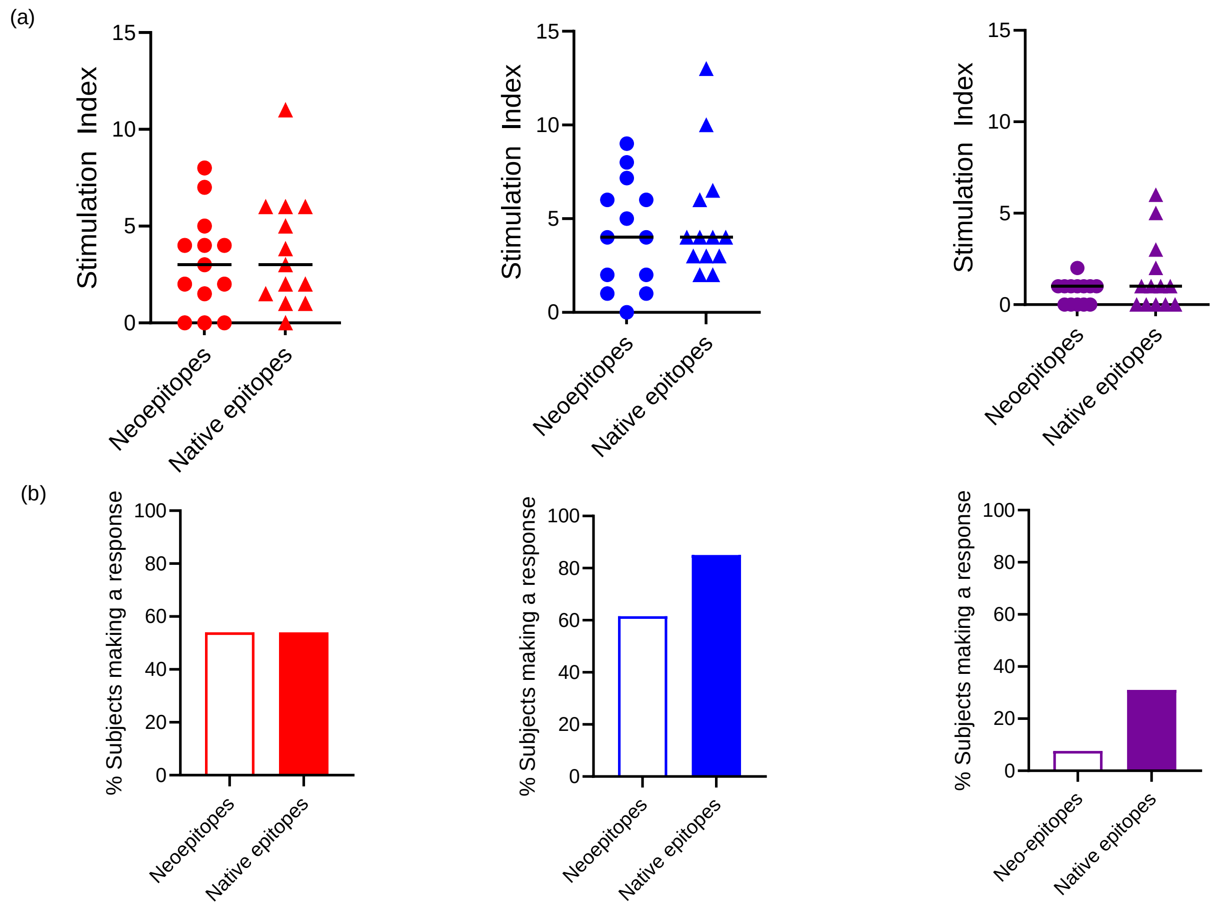

Supp. Figure 8(a): Magnitude of responses as depicted by SI for IFN-γ (left) IL-10 (centre) and IL17 (right) responses against pooled neo- and native epitopes in preclinical subjects.

Supp. Figure 8(b): Prevalence of IFN-γ (left), IL-10 (centre) and IL-17 (right) responses in preclinical subjects against pooled peptides of neo- (open bars) and native (filled bars) epitopes.

Supplementary Figure 9

(a)

(b)

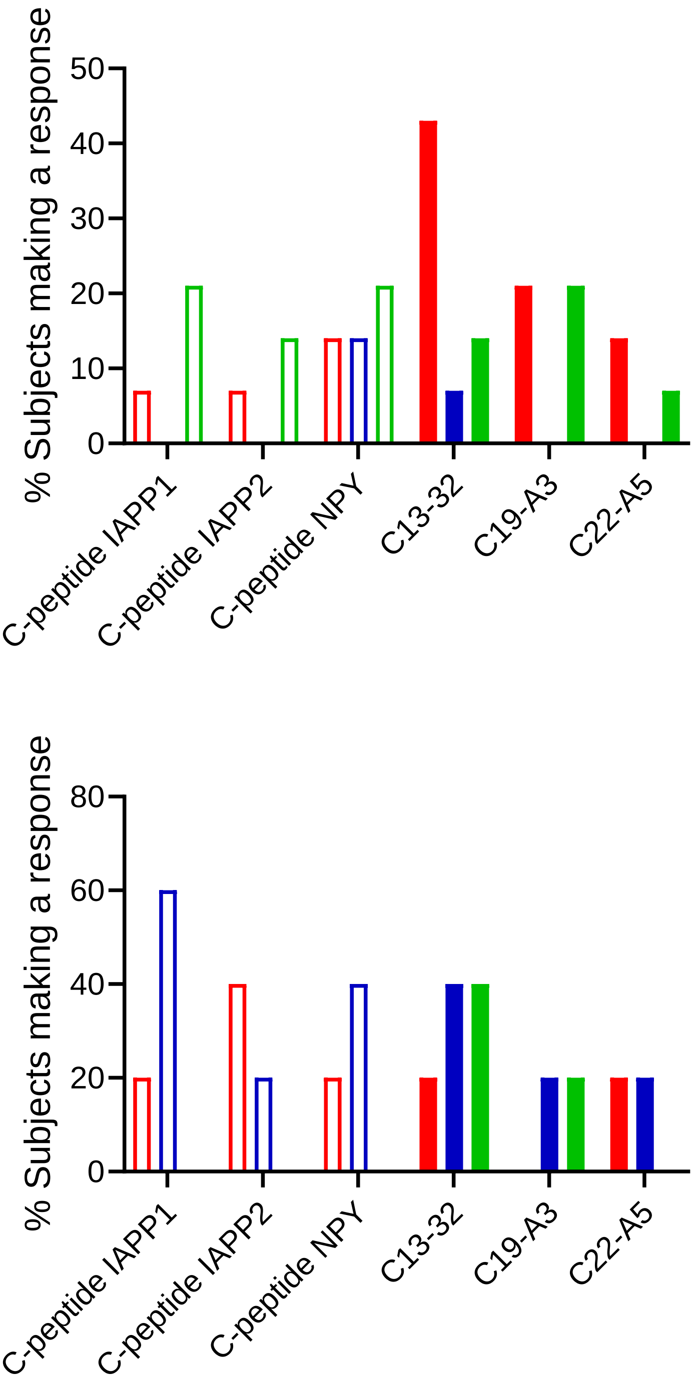

Supplementary Fig. 9: Prevalence of IFN-γ (red), IL-10 (blue) and IL-17 (green) responses in (a) children with type 1 diabetes and (b) preclinical children against peptides of neo- (open bars) and native (filled bars) epitopes.

Supplementary Figure 10

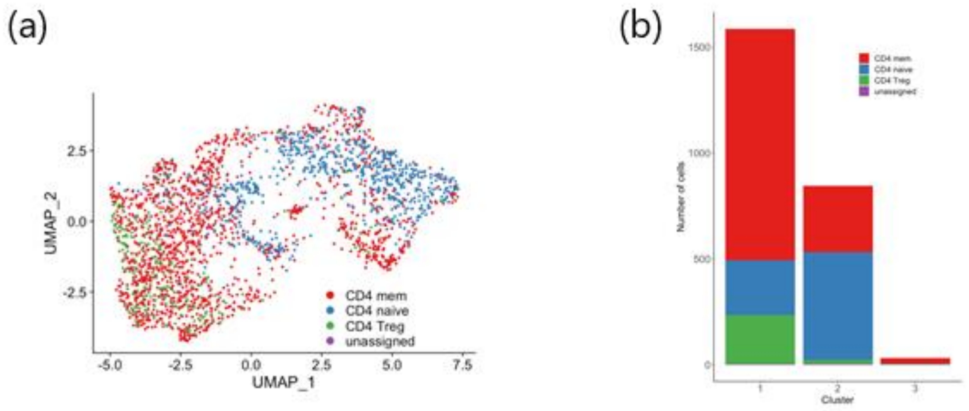

Supplementary Fig. 10: (a) UMAP segregates cells largely according to their reference-set inferred cell subtype (Individuals ND01 and ND02). (b) Clusters are dominated by memory (cluster 1) and naïve (cluster 2) cells, as inferred from a reference PBMC set.
