# Supplementary material for: Mapping T Cell Responses to Native and Neo-Islet Antigen Epitopes in at Risk and Type 1 Diabetes Subjects
Source: Front Immunol. 2021 Jun 25;12:675746. doi: 10.3389/fimmu.2021.675746 (PMC8274489; doi:10.3389/fimmu.2021.675746)
Supplement: Supplementary file 3 [file Table_1.docx]

**Supplementary Table 1:** Comparison of gene expression between native and neoepitope pools for subjects ND01 & ND02

| **p_val** | **avg_logFC** | **pct.1** | **pct.2** | **p_val_adj** | **clu** | **gene** |
| --- | --- | --- | --- | --- | --- | --- |
| 7.64E-05 | 1.097368985 | 0.107 | 0.035 | 0.970554283 | c_1 | IFNG |
| 3.40E-04 | 0.53280453 | 1 | 0.304 | 1 | c_2 | EBAG9 |
| 4.61E-04 | 0.53280453 | 0.778 | 0.174 | 1 | c_2 | ANK3 |
| 0.001445441 | 0.601797402 | 0.778 | 0.217 | 1 | c_2 | ZNF22 |
| 0.0022265 | 0.62461208 | 0.667 | 0.087 | 1 | c_2 | NME1 |
| 0.003784797 | 0.662016262 | 0.889 | 0.261 | 1 | c_2 | ZFR |
| 0.004522835 | 0.520534438 | 1 | 0.565 | 1 | c_2 | DHRS7 |
|  |  |  |  |  |  |  |
