# Supplementary material for: Mapping T Cell Responses to Native and Neo-Islet Antigen Epitopes in at Risk and Type 1 Diabetes Subjects
Source: Front Immunol. 2021 Jun 25;12:675746. doi: 10.3389/fimmu.2021.675746 (PMC8274489; doi:10.3389/fimmu.2021.675746)
Supplement: Supplementary file 4 [file Table_2.docx]

**Supplementary Table 2: T cell clonotypes against native or neoepitopes.**

| **Patient & Clonotype** | **TRA** | **TRB** | **Cells Native epitopes** | **Cells Neo-epitopes** |
| --- | --- | --- | --- | --- |
| ND01 14 | CAVKGNQGGKLIF | CASTRHESNQPQHF | 4 |  |
| ND01 41 | CAINSGYALNF: | CASMTSAYNEQFF |  | 4 |
| ND01 85 | CAVQRGNTPLVF | CASSLRTSGVYNEQFF |  | 4 |
| ND01 109 | CAGPGNSGYALNF | CASRPTVGWNTEAFF | 3 |  |
| ND01 114 | CAVDSNYQLIW | CASSMTSLWNEQFF | 3 |  |
| ND01 115 | CLLGGSQGNLIF  CAVGRSQGNLIF | CASSPGQQETQYF |  | 3 |
| ND01 135 | CAASKAGYSTLTF | CASRPGTSNRELFF |  | 3 |
| ND01 150 | CAVSGGSYIPTF | CASSLSTVGNTIYF | 3 |  |
| ND01 46 | CATDARGAGSYQLTF | CASSLGPATQETQYF | 3 |  |
| ND01 10/2974 | CAGETGANSKLTF  CAVRSNNAGNMLTF | CASSHLGAYEQYF | 4 |  |
| ND01 1068/63 | CALSVSGAGSYQLTFCAVSPSGGYQKVTF | CSVTTEVGSGANVLTF |  | 4 |
| ND01 127/3062 | CATRPRTDKLIF  CAVNAPVNGGATNKLIF | CASSPAGGSYNEQFF |  | 3 |
| ND02 7 | CAGGGEETSGSRLTF | CASSQPGHNNEQFF | 12 |  |
| ND02 24 | CAVRSEETSGSRLTF | CASSQVGHNEKLFF | 4 |  |
| ND02 71 | CAVRSEETSGSRLTF | CASSQAGHNNEQFF | 4 |  |
| ND02 86 | CAGRDDYKLSF | CASSLRGRQPQHF |  | 4 |
| ND02 134 | CAASYNQGGKLIF | CSVEKSPSELFF | 3 |  |
| ND02 155 | CAVAPQGGSEKLVF | CASSSYRAMNTEAFF | 3 |  |
| ND02 45 | CASPLRNQGGKLIF | CASSFYGNTEAFF | 3 |  |
| ND02 87 | CALTPNTGGFKTIF | CASSSRVLDSPLHF | 3 |  |
| ND02 93 | CAAGRTDS WGKLQF | CASSLTQVNEQFF | 3 |  |

Sequences highlighted in red correspond to those listed in the JDRF npod TCR database.
