# Supplementary material for: Mapping T Cell Responses to Native and Neo-Islet Antigen Epitopes in at Risk and Type 1 Diabetes Subjects
Source: Front Immunol. 2021 Jun 25;12:675746. doi: 10.3389/fimmu.2021.675746 (PMC8274489; doi:10.3389/fimmu.2021.675746)
Supplement: Supplementary file 5 [file Table_3.docx]

Supplementary Table 3: Prevalence of T cell responses to native or neoepitopes in subjects with and without HLA-DR4/DQ8.

|  | HLA-DR4/DQ8 | Non-HLA-DR4/DQ8 |
| --- | --- | --- |
| Native epitopes | 30% | 30% |
| Neoepitopes | 26% | 16% |
